# Identification of neutrophil β2-integrin LFA-1 as a potential mechanistic biomarker in ANCA-associated vasculitis via microarray and validation analyses

**DOI:** 10.1186/s13075-021-02510-1

**Published:** 2021-05-06

**Authors:** Kotaro Matsumoto, Takahiko Kurasawa, Keiko Yoshimoto, Katsuya Suzuki, Tsutomu Takeuchi

**Affiliations:** 1 Division of Rheumatology, Department of Internal Medicine, Keio University School of Medicine, 35 Shinanomachi, Shinjuku-ku, Tokyo Japan; 2 Department of Rheumatology and Clinical Immunology, Saitama Medical Center, Saitama Medical University, 1981 Kamoda, Kawagoe, Saitama Japan

**Keywords:** ANCA-associated vasculitis, Gene expression, β2-integrin, Lymphocyte function-associated antigen-1

## Abstract

**Background:**

Leukocyte activation by anti-neutrophil cytoplasmic antibody (ANCA) and the subsequent leukocyte–endothelium interaction play a key role in the development of endothelial damage in ANCA-associated vasculitis (AAV). In contrast to that of leukocyte activation, the exact role of the leukocyte–endothelium interaction via integrin remains unclear. Here, we performed microarray and validation analyses to explore association between the expression levels of lymphocyte function-associated antigen-1 (LFA-1) and the clinical characteristics of patients with AAV.

**Methods:**

We performed gene set enrichment analysis (GSEA) to identify the functional gene sets differentially expressed between patients with AAV and other types of vasculitis and the healthy controls (HCs). Flow cytometry was performed to validate the GSEA results. Treatment-naïve patients were monitored until 24 weeks of treatment. To examine the role of LFA-1 in the neutrophil–endothelium interaction, we performed a leukocyte adhesion and transmigration assay using peripheral blood and human umbilical vein endothelial cells (HUVECs).

**Results:**

GSEA revealed that the molecular pathways involving integrin-related genes were significantly upregulated in patients with AAV compared to that in patients with other types of vasculitis and the HCs. Flow cytometry revealed that the percentage of neutrophils expressing LFA-1 was significantly higher in patients with AAV than in those with large-vessel vasculitis or polyarteritis nodosa and the HCs. LFA-1 levels in the neutrophils were higher in patients with MPO-ANCA-positive expression than in those with a positive PR3-ANCA expression and correlated with the peripheral eosinophil count, serum rheumatoid factor titre, serum C-reactive protein levels, and the vasculitis activity score of systemic and chest components. After 24 weeks of treatment, including prednisolone, cyclophosphamide, rituximab, azathioprine, methotrexate, and/or tacrolimus, neutrophil LFA-1 expression remained high in the non-responder patients, but decreased in the responder patients. The in vitro assay showed that leukocyte migration toward HUVECs was dependent on the interaction between LFA-1 and intercellular adhesion molecule-1 (ICAM1); the migration of leukocytes was inhibited by blocking the adhesion of LFA-1 to ICAM1.

**Conclusions:**

The expression of LFA-1 in neutrophils is increased in patients with AAV. Neutrophil LFA-1 levels correlate with the clinical features of AAV. Inhibiting the adhesion of LFA-1 and ICAM1 impedes the neutrophil–endothelium interaction and may have a therapeutic role in AAV.

**Supplementary Information:**

The online version contains supplementary material available at 10.1186/s13075-021-02510-1.

## Background

Anti-neutrophil cytoplasmic antibody (ANCA)-associated vasculitis (AAV) is an autoimmune disease that affects small- to medium-sized blood vessels and includes microscopic polyangiitis (MPA), granulomatosis with polyangiitis (GPA), and eosinophilic granulomatosis with polyangiitis (EGPA) [1–3]. Neutrophils in the peripheral blood are elevated and play a central role in the pathogenesis of AAV [4–7]. Although the phenotypes and functional changes in the endothelial cells of AAV patients are not fully understood, an elevated proportion of circulating endothelial cells is associated with disease activity in AAV [8–10]. While vasculitis is associated with abnormalities in circulating immune cells and vascular endothelial cells [11–13], the exact role of the neutrophil–endothelium interaction in AAV remains unclear.

Activated leukocytes in the peripheral blood are known to display altered levels of surface molecules. For example, CD11b levels in neutrophils and monocytes are elevated, while CD62L levels in monocytes are decreased in patients with GPA [14–18]. ANCA can induce neutrophil activation via interaction with the αM (CD11b) and β2 (CD18) subunits of integrin Mac-1 [14, 15]. Granules from activated neutrophils and neutrophil extracellular traps stimulate endothelial reactive species and induce endothelial damage [19].

The leukocyte–endothelium interaction is essential for governing the movement of leukocytes toward the site of inflammation and regulating leukocyte recruitment. Lymphocyte function-associated antigen-1 (LFA-1) is an αL (CD11a) and β2 (CD18) integrin subunit, which interacts with intercellular adhesion molecule-1/2 (ICAM1/2) on endothelial cells [20–22]. We previously reported that LFA-1 is upregulated in patients with systemic lupus erythematosus with vasculitis [23]. However, the role of LFA-1 in AAV is not well understood.

AAV is primarily managed with cyclophosphamide- or rituximab-based treatments; however, close to 50% of patients experience disease relapses [24–26], and a therapeutic drug that can target neutrophil activation has not yet been developed. Considering the fact that the neutrophil–endothelium interaction is an essential process [27, 28], integrin-mediated neutrophil–endothelium adhesion may act as a promising therapeutic target in AAV.

In this study, we performed a microarray analysis and validation to identify the key molecules involved in integrin-mediated cell adhesion occurring in AAV. Subsequently, we identified LFA-1 and explored its role in AAV.

## Patients and methods

### Patients and healthy controls

**Fig. 1:**
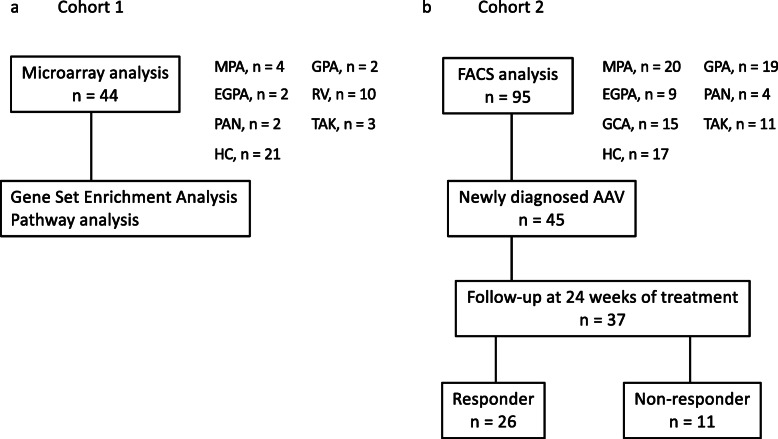
Study design and analytical strategy. **a** Microarray analysis and **b** fluorescence-activated cell sorting (FACS) analysis for validation. AAV, anti-neutrophil cytoplasmic antibody-associated vasculitis; MPA, microscopic polyangiitis; GPA, granulomatosis with polyangiitis; EGPA, eosinophilic granulomatosis with polyangiitis; RV, rheumatoid vasculitis; PAN, polyarteritis nodosa; TAK, Takayasu arteritis; HC, healthy control

To explore the key molecule associated with the clinical characteristics of patients with AAV, we first conducted a microarray analysis and analysed the data using the Gene Set Enrichment Analysis (GSEA) and a pathway analysis (Fig. 1a). Following this, a fluorescence-activated cell sorting (FACS) analysis was performed to validate the microarray results (Fig. 1b). Whole blood samples were collected from patients at the Keio University Hospital, between April 2008 and January 2021. Patients with MPA (*n* = 4), GPA (*n* = 2), EGPA (*n* = 2), rheumatoid vasculitis (RV; *n* = 10) [29], polyarteritis nodosa (PAN; *n* = 2) [30], and Takayasu arteritis (TAK; *n* = 3) [31], who met the respective international classification criteria, and 21 healthy controls (HCs) were enrolled in the microarray analysis. Additionally, active patients with AAV (total number = 48; MPA, *n* = 20; GPA, *n* = 19; EGPA, *n* = 9), LVV (total number = 26; TAK, *n* = 11; giant cell arteritis [32], *n* = 15), PAN (*n* = 4), and HCs (*n* = 17) were enrolled in the validation analysis. Patients with GPA were further compared between generalised (*n* = 15) and localised (*n* = 4) forms, as previously categorised [33]. We confirmed that the HCs did not report a history of any autoimmune disease, severe allergic disorder, malignancy, or infection.

This study was approved by the Institutional Review Board of Keio University School of Medicine (#20140335) and was conducted in compliance with the Declaration of Helsinki. Written informed consent was obtained from all participating individuals.

### Clinical assessment

Clinical information was obtained from patient records. Disease activity in vasculitis patients was recorded using the Birmingham Vasculitis Activity Score (BVAS) 2003, along with clinical signs [34]. Evidence of organ involvement (mucous membranes/eyes, ear, nose, throat, chest, kidney, and nervous system) and laboratory data, including the erythrocyte sedimentation rate, white blood cell count, haemoglobin level, platelet count, C-reactive protein (CRP) level, IgG level, ANCA positivity, ANCA titre, and rheumatoid factor (RF) levels, were assessed. Remission was defined as the absence of clinical signs in the disease activity, as indicated by a BVAS of 0 maintained for at least 2 months [26]. Patients who were treatment-naïve at the time of inclusion received treatment, including prednisolone, cyclophosphamide, rituximab, azathioprine, methotrexate, and/or tacrolimus, during the 24 weeks of follow-up evaluations. These patients were categorised into responder and non-responder groups according to whether they were in remission at 24 weeks of treatment.

### RNA extraction

Blood samples were collected in the PAXgene blood RNA tubes (PreAnalytiX, Hombrechtikon, Switzerland). Total RNA was extracted using the PAXgene blood RNA kit (PreAnalytiX) according to the manufacturer’s instructions. Total RNA quantity and quality were determined using a NanoDrop 1000 spectrophotometer (Thermo Fisher Scientific, Waltham, MA, USA) and an Agilent 2100 Bioanalyzer (Agilent Technologies, Santa Clara, CA, USA), respectively. All RNA samples met the following criteria: RNA integrity > 7 and optical density at 260/280 nm > 1.6.

### Microarray experiment

Cyanine 3-labelled complementary RNAs (cRNAs) were synthesised using the QuickAmp Labelling Kit (Agilent Technologies). The cRNAs were hybridised to the whole human genome at 65 °C for 17 h using Microarray 8 × 60 K v 2.0 (Agilent Technologies). After washing, the microarrays were scanned using an Agilent DNA microarray scanner (Agilent Technologies). The intensity value for each scanned feature was extracted using the Agilent Feature Extraction software (Agilent Technologies).

### GSEA and pathway analysis

We performed GSEA with v 2.1.0 (http://www.broadinstitute.org/gsea/index.jsp) using GeneSpring GX v 11.0.2 (Agilent technologies) to identify the functional gene sets that were differentially expressed among patients with AAV, disease controls, and HCs. Fold change (FC) was calculated to determine whether a set of genes showed statistically significant, concordant differences between two biological states. A two-sided unpaired Welch’s *t* test was performed for each pair of comparison groups, and adjusted *P* values were calculated using the Benjamini and Hochberg correction. Statistically significant changes in proteins or metabolites were selected using an adjusted *P* value < 0.05 and absolute FC > 1.3. Pathway analysis was performed using MAPPFinder (GenMAPP, www.genmapp.org).

### FACS analysis

We investigated the surface expression of the α- and β-integrin subunits on neutrophils, monocytes, and lymphocytes using FACS analysis. FACS analysis of the cells present in 20 μL of heparinised blood samples was performed. For gating purposes, the neutrophil population was verified by staining with an anti-CD16b-PE antibody (Clone CLB-gran11.5; BD Biosciences, San Jose, CA, USA). Monocytes and lymphocytes were gated using forward-and side-scatter. The antibodies used to probe surface molecules included anti-CD11a/CD18-Fluor488 (Clone M24; BioLegend, San Diego, CA, USA), anti-CD11a-PE-Cy7 (Clone HI111; BioLegend), anti-CD11b-FITC (Clone M1/70; BioLegend), anti-CD11c-VioBlue (Clone 4.9; BioLegend), and anti-CD18-APC (Clone TS1/18; BioLegend). Isotype-matched control IgGs for the corresponding antibodies were used as negative controls.

The fluorescence-activated cell sorting (FACS) analysis was conducted on an MACS Quant Analyser (Miltenyi Biotec, Auburn, CA, USA) using FlowJo v 10.1. (Tree Star, Ashland, OR, USA). Details of the gating strategy are provided in Supplementary Fig. 1. We collected clinical data and peripheral blood samples from patients with AAV until 24 weeks of treatment.

### In vitro leukocyte adhesion and transmigration assay

To examine the function of LFA-1 in the leukocyte–endothelium interaction, we performed a leukocyte adhesion and transmigration assay using human umbilical vein endothelial cells (HUVECs) and peripheral leukocytes from HCs, as previously described [35]. HUVECs were grown in EGM™-2 (Lonza, Basel, Switzerland) to 70–80% confluence and then treated with 10 ng/mL tumour necrosis factor (TNF)-α (PeproTech, Rocky Hill, NJ, USA) for 24 h to induce ICAM expression. Peripheral blood was used after haemolysis using HetaSep (Veritas, Tokyo, Japan). Subsequently, the Leuko Tracker solution (Cell Biolabs, San Diego, CA, USA) and 100 ng/mL of lipopolysaccharide (LPS)-pre-treated peripheral blood (1 × 10^5^ cells/well) were loaded into the upper chamber of Transwell inserts (3.0 μm pore size, 24-well plates; Corning, NY, USA). Recombinant interleukin (IL)-8 (10 ng/mL, PeproTech) and N-formyl-met-leu-phe (fMLP) (100 nM, Sigma-Aldrich, St Louis, MO, USA) were added to the lower compartment of each well as chemoattractants. Transmigrated cells were collected after co-incubation of leukocytes and HUVECs for 2 h and quantified by measuring the fluorescence intensity of Leuko Tracker solution-labelled leukocytes in the culture medium. The leukocyte transmigration assay was also conducted after treatment with 10 μg/mL of neutralising anti-LFA-1 antibody (Clone hu1124; Novus Biologicals, Littleton, CO, USA), anti-ICAM1 antibody (Clone 1A29; Thermo Fisher Scientific), and isotype-matched control IgG. The experiments were replicated in six healthy subjects.

### Statistical analysis

Continuous data are expressed as median and interquartile range (IQR), and categorical data are expressed as numbers and percentages. The Mann-Whitney *U* test was used to examine the differences between two groups, and the chi-squared test was used for nominal variables. Wilcoxon’s signed-rank test was used to compare paired samples. The spearman’s rank correlation coefficient was used for correlation analysis. Statistical significance was set at *P* < 0.05. All analyses were performed using the R statistics package (v 3.6.1; The R Foundation for Statistical Computing, Vienna, Austria), SPSS Statistics v 26.0 (IBM Corp., Armonk, NY, USA), and GraphPad Prism v 8.0 (GraphPad, La Jola, CA, USA).

## Results

### Identification of characteristic molecular profile for patients with AAV

**Fig. 2:**
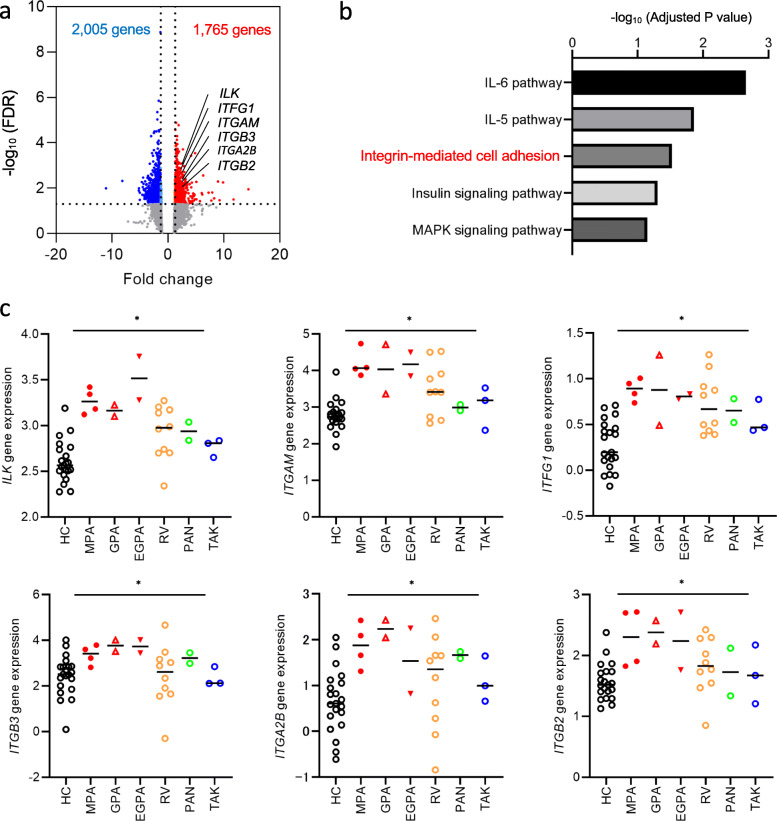
Upregulation of the molecules involved in integrin-mediated cell adhesion in AAV. **a** Volcano plot of 3770 differentially expressed genes between patients with AAV and the healthy controls (HCs) (fold change > 1.3, adjusted *P* < 0.05). **b** Top five pathways that were differentially regulated between patients with AAV and HCs. **c** *Z*-score variance of representative genes within each integrin family among patients with each type of vasculitis and the HCs, are shown. *P < 0.05 using the Kruskal-Wallis test. MPA, microscopic polyangiitis; GPA, granulomatosis with polyangiitis; EGPA, eosinophilic polyangiitis; RV, rheumatoid vasculitis; PAN, polyarteritis nodosa; TAK, Takayasu arteritis; *ILK*, integrin-linked kinase; *ITGAM*, integrin alpha M; *ITFG1*, integrin alpha FG-GAP repeat containing 1; *ITGB3*, integrin beta 3; *ITGA2B*, integrin alpha 2b; *ITGB2*, integrin beta 2

Baseline characteristics of the patients are shown in Table 1 and Supplementary Table 1. Among the 24 patients enrolled for transcriptome analysis, six were treatment-naïve and the remaining 18 were undergoing treatment (Supplementary Table 1). We used GSEA to identify the molecular biological features of each type of vasculitis. Table 2 shows the pathways that were upregulated and downregulated in AAV, RV, PAN, and TAK, compared to those in the HCs, using the permute *P* value < 0.05. At a significance threshold of the FDR adjusted *P* value < 0.05, and fold change > 1.3, we identified 3770 differentially expressed genes between patients with AAV and the HCs (Fig. 2a). Among the 1765 upregulated genes, the pathways related to IL-6 (adjusted *P* value = 0.0022), IL-5 (adjusted *P* value = 0.014), integrin-mediated cell adhesion (adjusted *P* value = 0.030), and insulin signalling (adjusted *P* value = 0.049) were significantly upregulated in patients with AAV compared to those in the HCs; the MAPK signalling pathway tended to be upregulated in patients with AAV (adjusted *P* value = 0.071) (Fig. 2b). As the leukocyte–endothelium interaction plays an important role in endothelial damage, we focused on integrin families. Regarding individual genes, the expression levels of genes associated with integrin-mediated cell adhesion (*ILK*, *ITGAM*, *ITFG1*, *ITGB3*, *ITGA2B*, and *ITGB2*) were higher in patients with AAV than in the disease controls and HCs (Fig. 2c).

**Table 1:** Baseline characteristics of patients, assessed using FACS analysis

Variable	AAV	LVV	PAN	HC
	*n* = 48	*n* = 26	*n* = 4	*n* = 17
Baseline characteristics				
Age, years	70 (57–80)	67 (47–72)	64 (48–72)	43 (32–56)
Male, *n* (%)	15 (31)	14 (54)	1 (25)	5 (29)
Race, Japanese, *n* (%)	48 (100)	26 (100)	4 (100)	17 (100)
MPA/GPA/EGPA, *n*	20/19/9	–	–	–
GCA/TAK, *n*	–	15/11	–	–
Newly diagnosed/major relapse, *n*	45/3	24/2	4/0	–
BVAS	12 (8–18)	3 (3–6)	15 (12–19)	–
Organ involvement				
Systemic, *n* (%)	34 (71)	23 (88)	2 (50)	–
Skin, *n* (%)	5 (10)	1 (4)	3 (75)	–
Mucous membranes/eyes, *n* (%)	5 (10)	0 (0)	0 (0)	–
Ear, nose, throat, *n* (%)	22 (46)	0 (0)	0 (0)	–
Chest, *n* (%)	28 (58)	0 (0)	1 (25)	–
Vascular, *n* (%)	3 (6)	9 (35)	1 (25)	–
Renal, *n* (%)	17 (35)	3 (12)	1 (25)	–
Nervous system, *n* (%)	20 (42)	0 (0)	3 (75)	–
Laboratory test				
ESR, mm/h	87 (40–121)	109 (59–122)	71 (33–112)	–
White blood cells, 10^3^ cells/μL	9.5 (7.0–13)	9.5 (7.0–14)	7.7 (6.6–9.1)	–
Neutrophils, 10^3^ cells/μL	6.2 (4.4–8.4)	5.4 (4.6–7.0)	6.1 (2.6–8.4)	–
Lymphocytes, 10^3^ cells/μL	1.5 (1.0–1.8)	1.4 (1.1–1.8)	1.1 (0.8–1.6)	–
Monocytes, cells/μL	414 (308–623)	460 (343–565)	444 (235–737)	–
Eosinophils, cells/μL	223 (116–814)	119 (67–192)	109 (7–755)	–
Haemoglobin, g/dL	11 (9.6–13)	11 (10–12)	12 (11–13)	–
Platelets, 10^4^ cells/μL	34 (25–41)	34 (29–42)	28 (18–36)	–
CRP, mg/dL	3.9 (0.7–8)	4.8 (2.4–6.5)	0.8 (0.3–5.2)	–
IgG, mg/dL	1648 (1120 − 1905)	1437 (1140–1690)	1325 (1124–2223)	–
MPO-ANCA-/PR3-ANCA-positive/ANCA-negative, *n*	34/8/6	1/0/25	0/0/4	–
MPO-ANCA titre, U/mL	41 (13–171), *n* = 34	28, *n* = 1	–	–
PR3-ANCA titre, U/mL	14 (10–39), *n* = 8	–	–	–
Rheumatoid factor positive, *n* (%)	37 (79)	2 (8)	1 (25)	–
Rheumatoid factor, IU/mL	65 (32–141)	29 (16–42)	41, *n* = 1	–

*AAV* ANCA-associated vasculitis, *MPA* microscopic polyangiitis, *GPA* granulomatosis with polyangiitis, *EGPA* eosinophilic granulomatosis with polyangiitis, *LVV* large-vessel vasculitis, *GCA* giant cell arteritis, *TAK* Takayasu arteritis, *PAN* polyarteritis nodosa, *BVAS* Birmingham vasculitis activity score, *ESR* erythrocyte sedimentation rate, *CRP* C-reactive protein, *MPO* myeloperoxidase, *PR3* proteinase 3

**Table 2:** Results of gene set enrichment analysis

	MAPP name	Number changed	Number measured	Number on MAPP	Permute *P*	Adjusted *P*
AAV > HC	IL-6 pathway	26	89	100	< 0.001	0.0022
	IL-5 pathway	25	63	69	< 0.001	0.014
	Integrin-mediated cell adhesion	24	79	99	< 0.001	0.030
	Insulin signalling pathway	38	128	159	< 0.001	0.049
	MAPK signalling pathway	36	129	162	0.001	0.071
	EGFR1 pathway	38	148	177	< 0.001	0.093
	Focal adhesion	34	132	187	< 0.001	0.13
	MAPK cascade	10	22	29	0.001	0.057
	B cell receptor pathway	36	146	158	0.001	0.23
	Regulation of actin cytoskeleton	25	95	146	0.005	0.33
	IL-3 pathway	24	93	101	0.008	0.39
	Pentose phosphate pathway	4	7	7	0.011	0.31
	Glycogen metabolism	10	31	36	0.011	0.59
	Proteasome degradation	16	59	61	0.014	0.62
	Cytochrome P450	9	28	71	0.016	0.65
	IL-1 pathway	11	35	38	0.017	0.57
	Eicosanoid synthesis	6	16	19	0.030	0.67
AAV < HC	Ribosomal proteins	27	86	88	< 0.001	< 0.001
	Homologous recombination	4	12	13	0.012	0.002
	Inflammatory response pathway	5	21	33	0.029	0.071
	Synthesis and degradation of ketone bodies	2	4	5	0.035	0.076
RV > HC	Proteasome degradation	9	59	61	< 0.001	0.020
	Prostaglandin synthesis regulation	4	20	31	0.003	0.099
	Apoptosis	6	76	82	0.019	0.80
	Nucleotide GPCRs	2	10	105	0.037	0.54
	Electron transport chain	6	87	105	0.042	0.93
RV < HC	Inflammatory response pathway	2	21	33	0.041	0.84
PAN > HC	Oxidative stress	3	18	28	0.010	0.31
	Nucleotide GPCRs	2	10	11	0.016	0.44
	Statin pathway	2	12	20	0.038	0.64
	Eicosanoid synthesis	2	16	19	0.048	0.85
PAN < HC	Wnt signalling and pluripotency	4	64	97	0.015	0.83
TAK > HC	Apoptosis	4	76	82	0.008	0.63
	Hypertrophy model	2	14	20	0.014	0.23
	GPCRDB class A Rhodopsin-like	4	115	262	0.049	0.96
TAK < HC	S1P signalling	2	20	25	0.038	0.67

*MAPP* microarray pathway profiler, *AAV* ANCA-associated vasculitis, *RV* rheumatoid vasculitis, *PAN* polyarteritis nodosa, *TAK* Takayasu arteritis, *HC* healthy controls

### Neutrophil LFA-1 upregulation in patients with AAV

Given that α- and β-integrins are preferentially expressed in human neutrophils, monocytes, and lymphocytes [20–22], we examined their surface expression using FACS analysis to confirm our GSEA results.

**Fig. 3:**
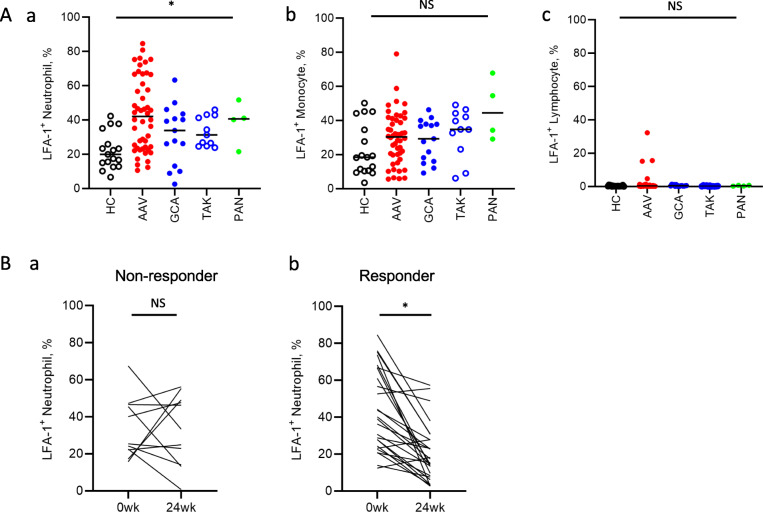
Upregulation of LFA-1 expression in the neutrophils of patients with AAV. (A) Expression of LFA-1 in (a) neutrophils, (b) monocytes, and (c) lymphocytes. (B) Temporal change in LFA-1 expression in the neutrophils of (a) non-responder and (b) responder AAV patients. *P < 0.05 using the (A) Kruskal-Wallis test or (B) Wilcoxon’s signed-rank test. LFA-1, lymphocyte function-associated antigen-1; AAV, ANCA-associated vasculitis; HC, healthy control; MPA, microscopic polyangiitis; GPA, granulomatosis with polyangiitis; EGPA, eosinophilic granulomatosis with polyangiitis; GCA, giant cell arteritis; TAK, Takayasu arteritis; wk., week; NS, not significant

In concordance with our GSEA results, the percentage of neutrophils expressing LFA-1 was higher in the neutrophils of patients with AAV than in those of the disease controls and HCs (HC vs. AAV vs. GCA vs. TAK vs. PAN; 44% vs. 34% vs. 31% vs. 41% vs. 20%, *P* = 0.0006) (Fig. 3A-a). In contrast, LFA-1 expression in the monocytes and lymphocytes was not significantly different between patients with AAV and LVV and the HCs (Fig. 3A-b and -c). Furthermore, CD11c expression in the monocytes was lower in patients with AAV than in the HCs (52% vs. 73%, *P* = 0.0007) (Supplementary Fig. 2C). In contrast, there was no significant difference in the expression of other integrins, including CD11a (Supplementary Fig. 2A), CD11b (Supplementary Fig. 2B), and CD18 (Supplementary Fig. 2D), between patients with AAV and the HCs.

### Temporal changes in LFA-1 expression in the responder and non-responder groups

Of the 45 treatment-naïve patients, 37 were monitored for at least 24 weeks following the procedure. These 37 patients were categorised as responders (*n* = 26) and non-responders (*n* = 11) based on the criteria described in the ‘Patients and methods’ section. LFA-1 expression in the neutrophils of non-responder patients was comparable at the onset and at 24 weeks of treatment (25% vs. 33%, *P* = 0.90) (Fig. 3B-a), but decreased in the responder patients (40% vs. 16%, *P* < 0.0001) during follow-up (Fig. 3B-b). The expression levels of neutrophil LFA-1 at baseline (40% vs. 25%, *P* = 0.11) and at 24 weeks of treatment (16% vs. 33%, *P* < 0.0001) were not significantly different between the responder and non-responder groups. Treatments received by the responder and non-responder patients from week 0 to week 24 are shown in Supplementary Table 2.

### Correlation between LFA-1 expression in neutrophils and clinical features

**Fig. 4:**
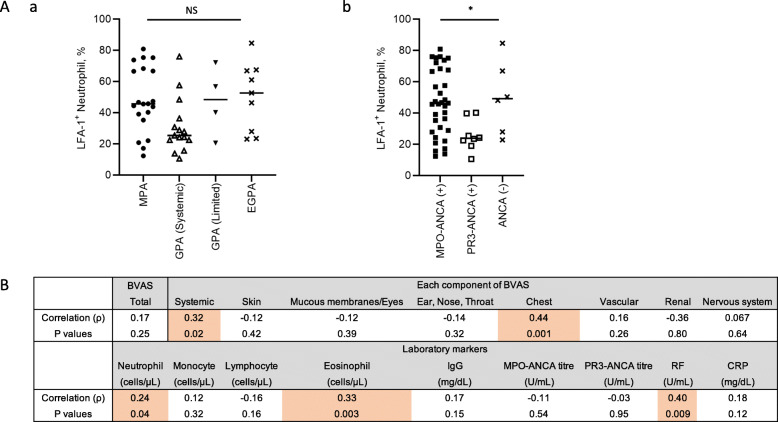
Correlation between LFA-1 expression in neutrophils and disease activity. (**A**) Expression of LFA-1 in neutrophils among patients with (a) different disease phenotype and (b) ANCA status. (**B**) Correlation analysis was conducted to determine the Spearman’s correlation coefficient between LFA-1 expression in neutrophils and disease activity. LFA-1, lymphocyte function-associated antigen-1; BVAS, Birmingham Vasculitis Activity Score; RF, rheumatoid factor; CRP, C-reactive protein

We compared the levels of LFA-1 with clinical phenotypes, which revealed no differences among patients with MPA, generalised GPA, localised GPA, and EGPA (Fig. 4A-a). The Kruskal-Wallis test and post-hoc test revealed that the levels of LFA-1 in neutrophils were comparable between patients with MPO-ANCA-positive and ANCA-negative expression, but were higher than those in patients with PR3-ANCA-positive expression (46% vs. 48% vs. 24%, *P* = 0.023) (Fig. 4A-b). We also examined the relationship between neutrophil LFA-1 expression levels and disease activity based on the BVAS and laboratory markers. Correlation analysis was conducted to determine the Spearman’s rank correlation coefficient between LFA-1 expression in neutrophils and disease activity (Fig. 4B). Correlation analysis showed that the BVAS scores in systemic (ρ = 0.32, *P* = 0.028) and chest (ρ = 0.48, *P* = 0.001) components, excluding total BVAS and BVAS scores in other components, were significantly correlated with the expression of LFA-1 in neutrophils. Moreover, LFA-1 expression in the neutrophils was significantly correlated with eosinophil count (ρ = 0.29, *P* = 0.048), serum RF level (ρ = 0.39, *P* = 0.018), and serum CRP level (ρ = 0.30, *P* = 0.038) in patients with AAV. These data indicate that elevated LFA-1 expression in neutrophils may be associated with systemic disease activity in AAV.

### Effects of LFA-1 and ICAM1 on the leukocyte–endothelium interaction

**Fig. 5:**
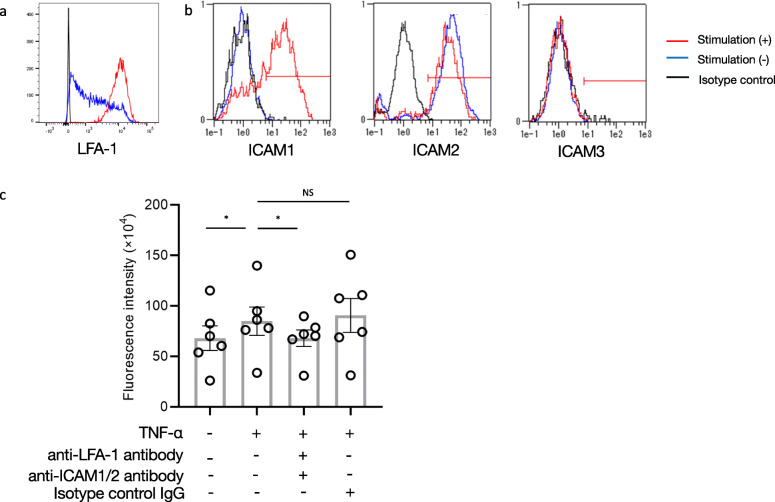
Effect of LFA-1 on leukocyte–endothelium transmigration. Representative findings from flow cytometry analysis evaluating **a** LFA-1 expression in leukocytes and **b** ICAM1–3 expression in HUVECs in the presence and absence of LPS and TNF-α stimulation, respectively. **c** The extent of cell transmigration, as measured by the fluorescence intensity of Leuko Tracker solution-labelled leukocytes from six healthy subjects. Leukocyte migration in the presence of anti-LFA-1 antibody, anti-ICAM1 antibody, and isotype-matched control IgG was monitored. *P < 0.05, using Wilcoxon’s signed-rank test. LFA-1, lymphocyte function-associated antigen-1; ICAM, intercellular adhesion molecule-1; HUVEC, human umbilical vein endothelial cell; LPS, lipopolysaccharide; TNF, tumour necrosis factor

We performed leukocyte adhesion and transmigration assays to study LFA-1 function in the leukocyte–endothelium interaction. To avoid neutrophil activation during cell isolation, leukocytes were obtained from whole blood samples. LFA-1 was upregulated in the leukocytes after treatment with LPS (Fig. 5a), and levels of ICAM1, but not ICAM2/3, were upregulated in the HUVECs after treatment with TNF-α (Fig. 5b). We co-incubated LPS-stimulated leukocytes and TNF-α-pre-treated HUVECs to examine leukocyte migration via the fluorescence intensity of Leuko Tracker solution-labelled leukocytes. We observed upregulation of LPS-pre-treated leukocyte migration toward TNF-α-pre-treated HUVECs on a Transwell plate (65 × 10^4^ vs. 82 × 10^4^, *P* = 0.031), suggesting that the leukocyte–endothelium interaction was dependent on the expression of LFA-1 in the leukocytes and that of ICAM1 in the HUVECs (Fig. 5c). Notably, pre-incubation of leukocytes with the anti-LFA-1 and anti-ICAM1 neutralising antibodies significantly suppressed the interaction between leukocytes and HUVECs (82 × 10^4^ vs. 71 × 10^4^, *P* = 0.031), whereas incubation with the isotype-matched control did not significantly affect the result (82 × 10^4^ vs. 91 × 10^4^, *P* = 0.44).

## Discussion

We performed a microarray analysis and GSEA to compare the molecular biological features of patients with AAV, the disease controls, and the HCs. Based on these analyses, we found that proteins of the integrin family were specifically upregulated in AAV. FACS analysis revealed that LFA-1 expression in neutrophils was significantly elevated in patients with AAV compared to that in patients with LVV and in the HCs. LFA-1 levels in the neutrophils of patients with AAV were significantly associated with systemic inflammatory markers such as eosinophil count, RF level, CRP level, and BVAS score of systemic and chest components. Interestingly, LFA-1 expression in neutrophils was higher in MPO-ANCA-positive patients with AAV than in those positive for PR3-ANCA. Considering that MPO-ANCA-positive patients with AAV are at a higher risk of death [36], mechanistic biomarkers would be helpful.

The neutrophil–endothelium interaction mainly occurs due to the binding of LFA-1 to its receptor ICAM1/2 on the endothelial cells [20–22]. As neutrophil–endothelium adhesion is an essential process in neutrophil-mediated endothelial damage, upregulation of LFA-1 in neutrophils during AAV can serve as a convenient biomarker. Although the regulatory mechanism of LFA-1 expression in AAV has not yet been sufficiently explained, cytokines, such as GM-CSF and TNF-α whose levels are elevated in patients with AAV [37], can induce LFA-1 upregulation [38].

β2-integrins are necessary for leukocytic inflammation. JAK2-V617F knock-in mice (JAK2^+/VF^), which possess a JAK2 activating mutation, reportedly showed increased β2-integrin activity in neutrophils [39]. A previous report showed that JAK2^+/VF^ mice had enhanced neutrophil–endothelium adhesion that led to pathologic thrombosis, which was inhibited by anti-β2 integrin neutralising antibodies [39]. Abnormal functioning of integrins in leukocytes, particularly neutrophils, may induce abnormal leukocyte–endothelium interactions, contributing to such pathological events.

Integrins have recently been identified as therapeutic targets for various inflammatory diseases [40]. For example, vedolizumab, a humanised monoclonal antibody that specifically recognises the α4 β7 heterodimer, is effective in the treatment of ulcerative colitis [41] and Crohn’s disease [42]. Furthermore, natalizumab, which targets the α4-integrin subunit, is effective for the treatment of multiple sclerosis [43] and Crohn’s disease [44]. Additionally, efalizumab, which targets LFA-1, is effective for the treatment of moderate-to-severe psoriasis [45]. However, once approved globally, it was discontinued due to an adverse event of progressive multifocal leukoencephalopathy reported in several cases [46]. In our study, LFA-1 expression in the neutrophils was upregulated in patients with AAV and was associated with systemic inflammation via the neutrophil–endothelium interaction. Thus, we believe that bispecific or multi-specific antibodies bridging LFA-1 and neutrophil surface molecules can recognise LFA-1 in neutrophils. Treatment with emicizumab, a bispecific antibody and a drug that specifically targets neutrophil LFA-1, showed a positive effect in patients with haemophilia A [47] and could be a safe and effective therapeutic alternative for AAV.

In contrast to LFA-1 expression in the neutrophils, CD11c expression in the monocytes is decreased in patients with AAV. We recently reported that CD14^++^ and CD16^+^, which are intermediate monocytes, play a substantial role in the development of AAV [7]. As CD11c is a characteristic of both intermediate and classical monocytes [48, 49], CD11c expression may be involved in monocyte migration to the local site of inflammation; however, further investigation is needed to confirm this hypothesis.

Our study had several limitations. First, the number of patients enrolled in this study may not be sufficient to clarify the specificity of the changes in LFA-1 expression in AAV. Second, the significance of LFA-1 as a biomarker is not sufficient to distinguish between the disease controls and HCs because they overlap among groups. Third, we did not study the phenotype of endothelial cells or platelets in this study, although several abnormalities have been reported in the pathogenesis of AAV [50].

## Conclusions

In conclusion, we demonstrated that LFA-1 upregulation may enhance endothelial damage in patients with AAV. To our best knowledge, this is the first study to identify the role of the neutrophil adhesion molecule LFA-1 in the pathogenesis of AAV. Further evidence on the mechanism of interaction between neutrophils and endothelial cells will be helpful in the identification of novel therapeutic targets for AAV treatment.

## Supplementary Information

**Additional file 1.**

## Data Availability

All data generated and analysed in this study are disclosed.

## Abbreviations

ANCA: Anti-neutrophil cytoplasmic antibody
AAV: ANCA-associated vasculitis
LFA-1: Lymphocyte function-associated antigen-1
GSEA: Gene set enrichment analysis
HC: Healthy control
HUVEC: Human umbilical vein endothelial cell
LVV: Large vessel vasculitis
MPA: Microscopic polyangiitis
GPA: Granulomatosis with polyangiitis
EGPA: Eosinophilic granulomatosis with polyangiitis
ICAM: Intercellular adhesion molecule
RV: Rheumatoid vasculitis
PAN: Polyarteritis nodosa
TAK: Takayasu arteritis
BVAS: Birmingham vasculitis activity score
CRP: C-reactive protein
RF: Rheumatoid factor
cRNA: Complementary RNA
FACS: Fluorescence-activated cell sorting
TNF: Tumour necrosis factor
LPS: Lipopolysaccharide
IL: Interleukin
fMLP: N-formyl-met-leu-phe
