# Supplementary material for: Identification of neutrophil β2-integrin LFA-1 as a potential mechanistic biomarker in ANCA-associated vasculitis via microarray and validation analyses
Source: Arthritis Res Ther. 2021 May 6;23:136. doi: 10.1186/s13075-021-02510-1 (PMC8101175; doi:10.1186/s13075-021-02510-1)
Supplement: Supplementary file 1 — Additional file 1. [file 13075_2021_2510_MOESM1_ESM.docx]

**SUPPLEMENTAL MATERIAL**

Supplementary Table S1. Characteristics of patients enrolled in microarray analysis

| Sample No. | Diagnosis | Age | Gender | Medication |
| --- | --- | --- | --- | --- |
| 1 | MPA | 67 | Male | PSL |
| 2 | MPA | 44 | Female | no |
| 3 | MPA | 73 | Male | PSL |
| 4 | MPA | 74 | Male | PSL |
| 5 | GPA | 68 | Female | PSL |
| 6 | GPA | 67 | Male | PSL, AZA |
| 7 | EGPA | 50 | Female | no |
| 8 | EGPA | 70 | Female | PSL |
| 9 | RV | 59 | Female | PSL, ADA |
| 10 | RV | 57 | Male | PSL |
| 11 | RV | 78 | Female | PSL, IVCY |
| 12 | RV | 51 | Female | PSL, AZA, SASP |
| 13 | RV | 80 | Female | TAC |
| 14 | RV | 71 | Female | PSL, AZA, MTX, IFX |
| 15 | RV | 58 | Female | PSL, BUC, MTX |
| 16 | RV | 77 | Female | no |
| 17 | RV | 70 | Male | TCZ |
| 18 | RV | 54 | Female | PSL, ADA |
| 19 | PAN | 75 | Female | PSL |
| 20 | PAN | 43 | Female | PSL, MTX |
| 21 | TAK | 45 | Female | no |
| 22 | TAK | 43 | Female | no |
| 23 | TAK | 45 | Female | no |

MPA, microscopic polyangiitis; GPA, granulomatosis with polyangiitis; EGPA, eosinophilic granulomatosis with polyangiitis; RV, rheumatoid vasculitis; PAN, polyarteritis nodosa; TAK, Takayasu arteritis; PSL, prednisolone; AZA, azathioprine; ADA, adalimumab; IVCY, intravenous cyclophosphamide; SASP, salazosulfapyridine; TAC, tacrolimus; MTX, methotrexate; IFX, infliximab; BUC, bucillamine

Supplementary Table S2. Treatment regimens of responder and non-responder patients

| Treatment | Responder | Non-responder |
| --- | --- | --- |
|  | n = 26 | n = 11 |
| PSL dose |  |  |
| Initial PSL dose, mg/day | 50 (40–60) | 45 (30–60) |
| PSL dose at week 4, mg/day | 25 (20–35) | 25 (20–35) |
| PSL dose at week 12, mg/day | 15 (10–17) | 15 (10–35) |
| PSL dose at week 24, mg/day | 10 (8–12) | 30 (10–35) |
| Immunosuppressive drugs during week 0-24 |  |  |
| IVCY, n (%) | 15 (47) | 4 (36) |
| Rituximab, n (%) | 6 (19) | 6 (55) |
| AZA, n (%) | 15 (47) | 3 (27) |
| MTX, n (%) | 2 (6) | 0 (0) |
| TAC, n (%) | 1 (3) | 1 (9) |

PSL, prednisolone; IVCY, intravenous cyclophosphamide; AZA, azathioprine; MTX, methotrexate; TAC, tacrolimus

Supplementary Fig. S1. Gating strategy for analysis of effect of integrins on leukocytes

Black, isotype-matched IgG control

Supplementary Fig. S2. Expression of integrin family proteins in patients with AAV and HCs

Expression of (A) CD11a, (B) CD11b (C) CD11c, and (D) CD18 in neutrophils, monocytes, and lymphocytes. *P < 0.05 using the Kruskal-Wallis test. HC, healthy control; AAV, anti-neutrophil cytoplasmic antibody-associated vasculitis; LVV, large vessel vasculitis; PAN, polyarteritis nodosa; NS, not significant
